# Impact of Metformin Therapy on Vitamin B12 Levels in Patients With Type 2 Diabetes Mellitus

**DOI:** 10.1002/edm2.70049

**Published:** 2025-04-14

**Authors:** Sahar H. Mahmoud, Fatma R. Elfargani, Nagwa Mohamed, Fiaza A. Alhamdi

**Affiliations:** ^1^ Department of Physiology, Faculty of Medicine Benghazi University Benghazi Libya

**Keywords:** DM, megaloblastic anaemia, metformin, T1DM, T2DM, vitamin B12 levels

## Abstract

**Introduction:**

Diabetes mellitus (DM) is a serious health condition affecting people worldwide with a high prevalence rate. DM is classified according to its aetiology into type 1 diabetes mellitus (T1DM) and type 2 diabetes mellitus (T2DM); several risk factors contribute to the development of T2DM, such as age, genetic predisposition and obesity. Metformin is a proven drug of choice, and it is used as a first‐line treatment for patients with T2DM to improve their glycaemic levels. Vitamin B12 is an important water‐soluble vitamin, and its deficiency can lead to megaloblastic anaemia.

**Material and Methods:**

A Cross‐sectional retrospective study was conducted on patients with T2DM in a Sedi Hussein diabetic polyclinic in Benghazi, Libya. The total sample was 381 patients: T1DM 15 (n), and T2DM 366 (n). T1DM was excluded from our results. Patients with T2DM were divided into two groups: the control group (not on metformin) consisted of 71 patients, and the group that received metformin (M group) consisted of 295 patients.

**Aims of the Study:**

To assess serum vitamin B12 levels in patients with T2DM and evaluate the prevalence of vitamin B12 deficiency among those receiving metformin treatment.

**Results:**

The M group significantly decreased vitamin B12 serum levels by 443.56 mg/mL versus the control group of 541.33 pg/mL (*p* = 0.003). The prevalence of patients with vitamin B12 deficiency under the metformin treatment was 23.84%. There was no significant difference in correlation between serum vitamin B12 levels and both doses of metformin and the duration of diabetes mellitus.

**Conclusion:**

Vitamin B12 was significantly decreased in patients with T2DM receiving metformin.

## Graphical abstract

The association between metformin treatment in type 2 DM and vitamin B12 deficiency.

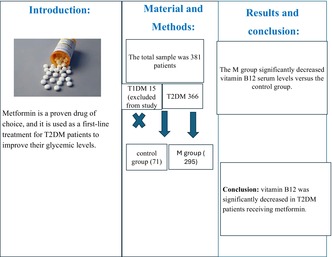

## 1 Introduction

Diabetes mellitus (DM) is a worldwide metabolic disorder with elevated blood glucose levels. It affects about 10.5% of people globally, and it increases morbidity and mortality rates. DM has two main types according to its aetiology: type 1 diabetes mellitus (T1DM) and type 2 diabetes mellitus (T2DM) [1, 2]. T2DM presents more in middle and older age groups than T1DM, which is prevalent in young age groups [3].

The cause of T1DM is a defect in pancreatic β cells to secrete insulin. Similarly, T2DM is also associated with insufficient insulin secretion. However, it additionally involves reduced sensitivity of the tissue's response to insulin [4].

T2DM accounts for about 85.0%–95.0% of all patients with diabetes and is more common than T1DM [5]. Globally, in a study published (2017) about 462 million (6.3%) people were affected. The rate is expected to increase in the upcoming years. T2DM causes a million deaths each year, making it one of the most common causes of mortality globally [6].

According to the International Diabetes Federation (IDF) in 2021, the prevalence of T2DM was about 12.2% in the Middle East and North Africa region. This marked increase is probably due to genetic predisposition, obesity and the lack of physical activity in the area [7]. Libya is one of the North African regions with a significant prevalence rate of T2DM, affecting more than 70.0% of the population. This rate is the highest in the region; this is related to poor eating habits and sedentary lifestyles [8].

T2DM has warning signs and symptoms, such as unexpected weight loss, fatigue, irritability, decreased vision and delayed wound healing. It also has classic symptoms, such as polydipsia, polyphagia and polyuria, which are also common in patients with T1DM [5].

Patients with T2DM should have good glycaemic control. It is estimated by the marker HbA1c, which reflects the blood glucose of a patient over a 2–3‐month duration [9]. Researchers found that a patient who has poor glycaemic control may develop complications like microvascular defects, such as retinopathy, nephropathy and neuropathy [10].

Therefore, early diagnosis and treatment of T2DM can prevent micro and macrovascular complications, or at least, delay the symptom's progression. The treatments used will slow the disease progression by reducing blood glucose levels, managing the patient's hypertension and dyslipidaemia, and lowering cardiovascular complications [11].

### 1.1 Metformin

It is an oral hypoglycaemic medication from the class Biguanide. Metformin is the initial drug of choice used to treat T2DM. It was proven by the FDA in 1994. In the UK it is one of the first‐line treatment drugs of choice and is commonly used following a 1998 prospective study that demonstrated the significant cardiovascular benefits of metformin [12]. Therefore, metformin is an important drug in improving glycaemic control, and it was proven to decrease mean HbA1c by 1.3% [13].

### 1.2 Vitamin B12

Vitamin B12 is an important water‐soluble vitamin. It plays an important role in haematopoiesis and neurological function [14]. Several studies conducted in the Middle East showed a relationship between vitamin B12 and metformin; one of these recent studies was conducted in Tripoli, Libya. The participants were 300: 150 had T2DM, and 150 were in the control group. The study's results revealed that serum levels of vitamin B12 deficiency were significantly decreased in patients with T2DM who were under metformin treatment compared with the control group. This is probably because Metformin inhibits calcium‐dependent channels in the ileum, which prevents the absorption of vitamin B12 [15].

The American Diabetes Association (ADA) recommends that patients with T2DM who take metformin as a treatment check their vitamin B12 serum levels regularly and take supplementary multivitamins to avoid vitamin B12 deficiency [16].

### 1.3 Literature Review

Metformin is administered orally in a tablet form, typically at a dose of 500 to 1000 mg usually taken twice daily. Metformin's mechanism of action is to increase the hepatic protein kinase activity, thus reducing both the hepatic gluconeogenesis and lipogenesis and enhancing glucose uptake by tissues through activation of the glucose 4 transporter (GLUT4) in the skeletal muscles and adipose tissue. Therefore, metformin is important in improving glycaemic control in the body [17, 18].

A cross‐sectional study by Jinwoon Kim et al. involving 1111 patients with T2DM who took metformin for at least 6 months, found that about 22.2% of the patients had serum vitamin B12 deficiency [19]. In addition, another study conducted in the Middle East, particularly in Saudi Arabia, showed that vitamin B12 deficiency and borderline deficiency were strongly associated with Metformin type of treatment. It also showed that patients who were under higher doses of metformin, more than 1000 mg, had lower levels of vitamin B12 [20]. About 69.6% of the patients with T2DM in Tripoli, Libya, had uncontrolled and poor glycaemic control in the follow‐up, which may predispose the patient to long‐term complications [21]. Long‐term complications are more prevalent in patients with T2DM, especially if their condition is poorly managed. In addition, patients suffering from T2DM may suffer from cardiovascular morbidity and mortality when compared with non‐diabetic patients, and an increase in cardiovascular death rate by 4.4‐fold [22].

Vitamin B12, or cobalamin, is a water‐soluble vitamin found in various foods, such as meat, fish, eggs and dairy products. It is important for DNA synthesis, myelination and the function of the central nervous system [14].

Patients with serum B12 above 300 pg/mL are considered normal, a range between 200 pg/mL and 300 pg/mL is regarded as a borderline deficiency, whereas patients with serum B12 below 200 pg/mL are considered deficient [23]. Vitamin B12 works as a cofactor for the enzyme methionine synthase. This enzyme facilitates the conversion of homocysteine into methionine, which is important in DNA synthesis. In vitamin B12 deficiency, homocysteine cannot convert into methionine, slowing DNA synthesis, which leads to megaloblastic anaemia [24].

The intrinsic factor is a glycoprotein produced by stomach parietal cells. It binds to vitamin B12 to be absorbed later in the distal ileum. Vitamin B12 deficiency occurs in patients undergoing gastrectomy, resection of the terminal ileum or gastric bypass, and it is also seen in patients who suffer from Crohn's disease or celiac disease due to a decrease in intrinsic factor production [24].

Vitamin B12 serves as a cofactor of the enzyme methyl malonyl‐coA mutase, which is responsible for converting methyl malonyl‐coA into succinyl‐coA. In individuals with vitamin B12 deficiency, the rise in methylmalonic acid (MMA) and homocysteine levels can result in myelin damage, leading to neuropathy. As a result, a lack of vitamin B12 can lead to megaloblastic anaemia and neurological dysfunction [24, 25].

Vitamin B12 deficiency is quite prevalent among older adults because of their reduced ability to absorb this vitamin from food. In a retrospective study in 1996 in elderly patients aged 65 and older, 509 patients had T2DM, and 188 of them received metformin. The findings revealed that 53.2% of patients on metformin had vitamin B12 deficiency compared with 31.0% of patients with diabetes who were under other diabetes drugs to treat T2DM, such as sulfonylurea, for example, glimepiride and gliclazide, and thiazolidinediones, for example, rosiglitazone and pioglitazone [26].

Screening tests are important to diagnose patients suffering from T2DM. The tests include fasting blood sugar, glycated haemoglobin (HbA1c), random plasma sugar and oral glucose tolerance test (OGTT). The positive screening test is considered positive if fasting blood glucose is ≥ 126 mg/dL, HbA1c is > 6.5%, random blood sugar is ≥ 200 mg/dL, and OGTT after 2 h from 75 mg glucose load is ≥ 200 mg/dL. These tests are used to determine the patient's glycaemic control, which could be either good or poor [9].

## 2 The Aims of the Study

1. Determine the serum level of vitamin B12 in patients with type T2DM.
2. Prevalence of patients under metformin who have vitamin B12 deficiency.
3. The correlation between vitamin B12 deficiency and metformin dose and duration of DM.

## 3 Material and Methods

### 3.1 Patients Sample

A cross‐sectional retrospective study was conducted in the biggest diabetic polyclinic in Benghazi‐Libya from 1 January 2022 to 1 January 2024. The total sample was 381 patients, chosen by simple random sampling method: T1DM 15 (n) (3.9%) and T2DM 366 (n) (96.1%).

For patients with T2DM, 145 were males (39.6%), and 221 (60.4%) were females. The participants' ages varied from 12 to 80. The prevalence of T2DM under metformin treatment is 80.6%, and for patients who are not under metformin, it is 19.4%.

The sample was divided into two categories: the first group (metformin‐treated group [M group]) included 295 patients with T2DM who had been treated with metformin for at least 1 year. The control group comprised 71 patients with T2DM who were on alternative treatments, including insulin, linagliptin and dapagliflozin.

The exclusion criteria were patients diagnosed with T1DM, T2DM, patients who suffer from malabsorption syndrome, patients who have undergone gastric bypass surgery and other types of anaemia, such as pernicious, aplastic and haemolytic anaemia.

The Ethical Committees approved this study.

### 3.2 Data Collection

All patients' records were gathered, and a pre‐structured questionnaire was created. The information obtained included the patient's age, gender, duration of diabetes mellitus, metformin dosage, serum vitamin B12 levels and HbA1c levels. All the collected data were incorporated into the questionnaire. Additionally, mean corpuscular volume (MCV) levels were collected to determine whether patients had megaloblastic anaemia.

In this sample, the metformin doses given to the patients were 500 mg, 850 mg and 1000 mg. The serum vitamin B12 levels in patients undergoing treatment with metformin were evaluated to determine whether their levels were reduced or at borderline values to compare them with the levels found in the control group.

Patients with serum B12 above 300 pg/mL were considered normal; a range between 200 pg/mL and 300 pg/mL was regarded as a borderline deficiency, whereas patients with serum B12 below 200 pg/mL were considered deficient.

### 3.3 Statistical Analysis

The SPSS (Statistical Package for Social Sciences, version 26) was used in the statistical analysis. To assess continuous variables, an independent sample *t*‐test is used. All collected quantitative data are expressed as mean ± S.D. Pearson's correlation coefficient test was also used to estimate the correlation between the factors. The ‘*p* value’ of < 0.05 is regarded as statistically significant.

## 4 The Results

The overall sample comprised 381 patients, with 15 patients having T1DM, representing (3.9%) of the total, and 366 patients having T2DM, accounting for (96.1%). The prevalence of T2DM under metformin treatment is 80.6%, and in patients who are not under metformin, it is 19.4%.

**FIGURE 1:**
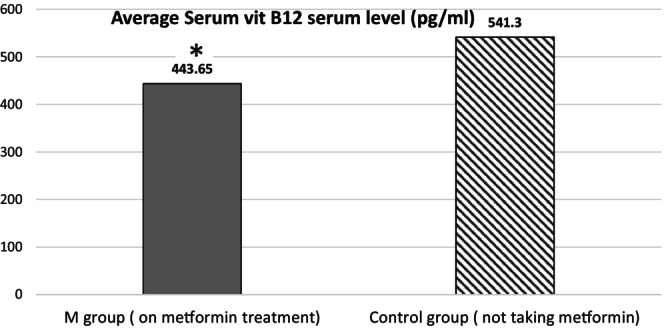
Average vitamin B12 serum level in both groups measured in picogram/mL. *p < 0.05.

The average age in T2DM was 50.79 ± 12.63. The average body mass index (BMI) of patients was 32.66 ± 8.45, the average HbA1c was 8.8% ± 3.9%. The average Hb of patients was 13.34 ± 1.84, the average MCV was 81.95 ± 7.88. The average vitamin B12 serum level is 461.54 ± 242.17, and the average years of DM were 6.54 ± 7.05 years (Tables 1, 2, 3, 4, 5, Figure 1).

**TABLE 1:** Number and percentage of females and males in each group.

	M Group	Control group
Male (*n*)	117	28
Female (*n*)	178	43
Total (*n*)	295	71
Male %	39.66	39.43
Female %	60.33	60.56

**TABLE 2:** Total number of patients and the percentage of patients with serum vitamin B12 levels in both groups.

	Severe deficiency	Borderline deficiency	Normal	Total
M Group (n)	9	58	214	281
Control group (n)	3	6	54	63
Total (*n*)	12	64	268	344
M group %	3.20	20.64	76.15	100
Control Group %	4.76	9.52	85.71	100

**TABLE 3:** Age groups and percentage of vitamin B12 levels in both groups (control and M).

Years of age	Severe deficiency %	Borderline deficiency%	Normal %
*Control group*			
(10–30) y	0	0	100
(30–50) y	6.67	16.67	76.67
(50–70) y	4.35	0	95.65
> 70 y	0	11.11	88.89
*M group*			
(10–30) y	0	15.38	84.62
(30–50) y	3.7	23.15	73.15
(50–70) y	2.78	19.44	77.78
> 70 y	6.25	18.75	75

**TABLE 4:** Relation in percentage between metformin dose and vitamin B12 deficiency in (M group).

%	500 mg	850 mg	1000 mg
Severe deficiency	3.03	3.88	3.03
Borderline deficiency	24.24	19.44	21.21
Normal	72.72	76.66	75.75

**TABLE 5:** The relation between the M group and the control group in vitamin B12, MCV, HbA1c%, BMI, and duration of diabetes mellitus.

	Control group	M group	*p*
B12	541.33 ± 283.65	443.56 ± 227.34	0.003*
MCV	83.33 ± 5.79	82.16 ± 6.67	0.44
HbA1c %	9.8 ± 7.9	8.6 ± 2.0	0.004*
BMI	32.12 ± 9.21	32.79 ± 8.27	0.8
Diabetes mellitus duration	6.39 ± 7.56	6.57 ± 6.92	0.39

* *p* < 0.05.

- The patients on metformin with vitamin B12 deficiency were in total 23.84% (*n* = 67 out of 281). At the same time, only 14.28% (*n* = 9 out of 63) of patients in the control group had vitamin B12 deficiency.
- The average serum vitamin B12 level in the M group was 443.65 ± 227.34 (mean ± S.D.). At the same time, the control group's average serum vitamin B12 level was 541.44 ± 283.65 (mean ± SD). ‘*p* value’ was 0.003.
- The most significant borderline deficiency is observed in individuals aged 30 to 50, with a rate of 23.15%.
- The Pearson correlation test: The correlation between the total serum vitamin B12 and MCV (*p* = 0.68), the doses of metformin (*p* = 0.9), and the duration of diabetes Mellitus (*p* = 0.7).

## 5 Discussion

The findings of this study reinforce the growing body of evidence linking metformin use with reduced serum vitamin B12 levels in patients with T2DM. The mean age group of the participants was 50.79 ± 12.63 years, which is consistent with findings from the study by Raizada et al. where the mean age was reported as 50.1 ± 11.5 years [27]. Such age distribution similarities emphasise our findings' relevance to broader populations of middle‐aged adults with T2DM [13].

Our data revealed that the prevalence of vitamin B12 deficiency in the M group was 23.84%, which aligns with previous studies, such as Kim et al.'s cross‐sectional study, which reported a similar prevalence of 22.2% among long‐term metformin users [19].

Additionally, borderline vitamin B12 deficiency was found in 20.64% of the M group, corroborating studies that have identified a comparable proportion of patients with borderline levels with 18.0% in one study and 20.6% in another [28, 29].

Several proposed mechanisms explain how metformin interferes with vitamin B12 absorption. Metformin appears to reduce the calcium‐dependent channels that facilitate the binding of the intrinsic factor–vitamin B12 complex to cubilin receptors in the terminal ileum, thereby impeding absorption [30]. Furthermore, metformin may reduce intrinsic factor secretion from gastric parietal cells, further contributing to diminished absorption [30]. This multifactorial interference highlights the need for regular monitoring of vitamin B12 levels in metformin‐treated patients, as recommended by the ADA [16].

In our study, the M group exhibited a significantly lower average serum vitamin B12 level (443.65 ± 227.34 pg/mL) compared with the control group (541.44 ± 283.65 pg/mL, *p* = 0.003). These findings align with research conducted in Tripoli, Libya, where metformin‐treated patients had significantly lower serum vitamin B12 levels compared with controls (216.6 pg/mL vs. 555.1 pg/mL) [15]. This significant reduction underscores the need for heightened awareness and proactive management of vitamin B12 deficiency in patients with T2DM on metformin therapy.

Interestingly, no statistically significant correlation was observed between vitamin B12 levels and either the metformin dose or the duration of diabetes mellitus in our study. This finding is consistent with the results of Almatrafi et al. who also found no significant association between these variables in a Saudi Arabian cohort [16]. This suggests that even low doses of metformin or shorter treatment durations may pose a risk for vitamin B12 deficiency, emphasising the importance of routine monitoring irrespective of dose or treatment duration.

Our data showed that borderline vitamin B12 deficiency was most prevalent in individuals aged 30–50 (23.15%), whereas severe deficiency was highest in those over 70 years (6.3%). Older adults are more prone to vitamin B12 deficiency because of diminished intrinsic factor production and decreased efficiency of vitamin B12 absorption. These findings align with Wong et al.'s study, which found a higher deficiency prevalence in older populations [31].

Regarding the impact of metformin dose in our study, borderline vitamin B12 deficiency occurred across all metformin dose groups (500 mg, 850 mg and 1000 mg daily) at rates of 24.24%, 19.44% and 21.21%, respectively. These results are consistent with a study reporting vitamin B12 deficiency in patients taking a broad range of metformin doses, from 500 mg to 2700 mg daily, over durations spanning 1–10 years. This highlights the progressive nature of the deficiency, suggesting that both dose and duration cumulatively affect vitamin B12 levels [32].

The absence of significant differences in MCV and BMI between the M and the control groups in our study further suggests that vitamin B12 deficiency can occur subclinically, without overt haematological manifestations. These findings are supported by Al‐Fawaeir et al. who also reported no significant changes in MCV or BMI among patients with vitamin B12‐deficiency [29].

However, the significantly lower HbA1c levels in the M group (*p* = 0.004) highlight metformin's efficacy in improving glycaemic control, as previously demonstrated in a randomised controlled study by González‐Ortiz et al. which reported a marked reduction in HbA1c levels after 2 months of metformin therapy compared with the control group [33]. These findings reaffirm metformin's role as a cornerstone in managing hyperglycaemia in patients with T2DM, effectively lowering HbA1c by an average of 1.3% [13].

Given the significant reduction in vitamin B12 levels among metformin users, we recommend annual screening for vitamin B12 deficiency in patients with T2DM on metformin therapy. Early detection and timely supplementation with vitamin B12, including intramuscular injections, when necessary, are critical to preventing complications, such as megaloblastic anaemia and neurological deficits. Long‐term prospective studies with larger sample sizes are needed to better elucidate the relationship between metformin dose, treatment duration, and the progression of vitamin B12 deficiency.

### 5.1 Study Limitations

The study was done retrospectively and essential data, such as the patient's duration of metformin usage could not be obtained from the patient's records. The number of participants was limited, especially the control group. Additionally, the use of methylmalonic acid to assess serum vitamin B12 levels was challenging because of cost‐effectiveness reasons.

## 6 Conclusion

This study highlights the significant reduction in serum vitamin B12 levels among patients with T2DM receiving metformin therapy compared with those not on metformin. The prevalence of vitamin B12 deficiency was notably higher in the metformin‐treated group, with no clear correlation observed between deficiency and metformin dose or duration of diabetes. These findings emphasise the critical need for routine monitoring of vitamin B12 levels in patients undergoing metformin therapy to detect and manage deficiencies early. Furthermore, the study underlines the efficacy of metformin in improving glycaemic control, evidenced by significantly lower HbA1c levels in the metformin group. Future research should focus on prospective studies with larger cohorts to further understand the long‐term implications of metformin use on vitamin B12 levels and overall patient health.

## Author Contributions

All authors contributed equally to the study.

## Disclosure

All authors declare that this research paper titled (Impact of Metformin Therapy on Vitamin B12 Levels in Patients with Type 2 Diabetes Mellitus) is entirely our work. We have cited all sources in this paper and have not plagiarised any material. Also, all data are available.

## Ethics Statement

This study was approved by (the Training and Development National Center for Diagnosis and Treatment of Diabetes in the Eastern Region/2024–29) and performed under the ethics standard written by the Helsinki Declaration 1964.

## Consent

All patients gave consent to participate in this study and to publish. If participants were under 18 years old, their parents gave informed consent.

## Conflicts of Interest

The authors declare no conflicts of interest.

## Data Availability

The authors confirm that the data supporting the paper is available, and all the data cited in our paper can be accessed.
